# Exploring the development, evaluation and implementation of complex health interventions to prevent early childhood caries in preschool children: A scoping review protocol

**DOI:** 10.1371/journal.pone.0275501

**Published:** 2022-10-10

**Authors:** Peggy C. J. M. van Spreuwel, Milou L. C. Munk-Overkamp, Linda C. Smit, Kirsten A. Ziesemer, Cor van Loveren, Geert J. M. G. van der Heijden, Josef J. M. Bruers, Katarina Jerković-Ćosić

**Affiliations:** 1 HU University of Applied Science, Research group Innovation in Preventive Healthcare, Utrecht, The Netherlands; 2 Oral Public Health Department, Academic Centre for Dentistry Amsterdam (ACTA), University of Amsterdam and Vrije Universiteit Amsterdam, Amsterdam, The Netherlands; 3 Royal Dutch Dental Association (KNMT), Utrecht, The Netherlands; 4 HU University of Applied Science, Research Centre for Healthy and Sustainable Living, Utrecht, The Netherlands; 5 Medical Library, VU University, Amsterdam, The Netherlands; 6 Academic Centre for Dentistry Amsterdam (ACTA), University of Amsterdam and VU University, Amsterdam, The Netherlands; University of Catanzaro, ITALY

## Abstract

**Objective:**

This scoping review aims to identify complex health interventions (CHI’s) to prevent early childhood caries (ECC), explore the level of complexity of the identified CHI’s, and explore the details of their development, evaluation, and implementation.

**Introduction:**

Many interventions to prevent ECC have multiple interacting components and can be seen as CHI’s. Recent reviews on these interventions have found inconclusive effects, which may be due to differences in the development, evaluation, and implementation of CHI’s.

**Inclusion criteria:**

This scoping review will consider clinical trials reporting CHI’s to prevent ECC that starts during pregnancy or in the first year of life. Studies in the English language will be included regardless of the country of origin, sociocultural setting, or context.

**Methods:**

This review will follow the Joanna Briggs Institute methodology for scoping reviews. An initial search of PubMed identified keywords and Medical Subject Headings terms. A second search of PubMed, Embase, Clarivate Analytics/Web of Science Core Collection, ClinicalTrials.gov, and the Wiley/Cochrane Library will follow. Two independent reviewers will perform title and abstract screening, retrieve and review full-text studies, and extract data. The reference lists of all included sources will be screened for additional CHI’s or relevant publications about a specific CHI. Data charting will be utilised based on study characteristics and intervention complexity. A 39-item instrument will be used to explore the details in the description of the CHI’s development, evaluation, and implementation. The results will be presented in tables, visual outputs, and a narrative summary in response to the review questions.

**Discussion:**

The proposed review will generate evidence which may provide a direction for the future design of studies on CHI’s to prevent ECC and more complete information for those who want to adopt successful interventions to prevent ECC.

## Introduction

Early childhood caries (ECC) is defined as the presence of a primary tooth with one or more carious (non-cavitated or cavitated lesions), missing (due to caries), or filled surfaces in a child under the age of six years [1]. It is considered one of the most prevalent diseases in childhood, affecting almost half of preschool children worldwide [2]. ECC consequences often include a higher risk of new carious lesions in primary and permanent dentition, hospitalisations and emergency room visits, high treatment costs, loss of school days, diminished ability to learn, and diminished oral health-related quality of life for both children and parents [3]. Literature on ECC preventive strategies assumes that interventions initiated during pregnancy or within the first year of life have a good chance of success [4–6]. However, recently published reviews have concluded that evidence on the effectiveness of early interventions to prevent ECC is inconclusive and that well-designed studies that include more children are needed [7, 8]. This means that studies should take into account the fact that even simple behavioural interventions for the prevention of ECC are often influenced by complex interplays of individual characteristics, social determinants, the health care delivery system, and the interventions themselves and therefore need to be treated as a complex health intervention (CHI’s) [9].

A CHI is characterised by multiple components (*intervention complexity*), complicated/multiple causal pathways, feedback loops, synergies, mediators, and moderators of effect (pathway complexity). In addition, they may have *population complexity* (target multiple participants, groups, or organisational levels), *implementation complexity* (require multifaceted adoption, uptake, or integration strategies), and/or *contextual complexity* (work in a dynamic multidimensional environment) [9]. Due to interacting mechanisms and components, contradictory effects are often found when a CHI is implemented under various circumstances. Therefore, evaluating the effects of CHI’s is challenging, and a specific multifaceted approach is required. Recent systematic reviews on this topic have included studies that meet the definition of CHI but are relatively straightforward with a classic reductive approach that is efficacy-focused without considering the complex interplays of an intervention [7, 8]. Instead of focusing only on the effectiveness of interventions, more emphasis should be placed on “how”, “for whom”, and “under what conditions an intervention can work” [9]. For those interested in adopting published interventions from reviews, more details are necessary to understand whether an intervention is feasible and which components are likely to work in their context, with their population and at what cost [9].

Since the introduction of the Medical Research Council framework in 2000 [10], there has been increasing interest in developing and evaluating CHI’s. In the meantime, several frameworks and guidelines have become available for the development, evaluation, implementation and reporting of CHI’s to enhance replication and reduce research waste [11–13]. While the field of CHI matures, this is still a relatively unknown domain in preventive oral health care. Given the inconclusive findings and recommendations of recent reviews, it would be highly relevant to assess the details of the development, evaluation, and implementation of interventions to prevent ECC and provide a direction for future study designs. A preliminary search of PubMed, Embase, PROSPERO, JBI Evidence Synthesis, and Open Science Framework revealed no current or underway systematic reviews or scoping reviews regarding the prevention of ECC that combine insights from frameworks and guidelines on the development, evaluation, implementation, and careful reporting of interventions. Therefore this scoping review aims to map CHI’s to prevent ECC that starts during pregnancy or in the first year of life, explore its complexity and the description of relevant aspects of the development, evaluation, and implementation. Outcomes of this scoping review may provide a direction for better design of studies on complex health interventions to prevent ECC and complete information for those who want to adopt successful interventions.

## Material and methods

The proposed scoping review will be conducted following the Joanna Briggs Institute (JBI) methodology for scoping reviews and reported using the Preferred Reporting Items for Systematic Reviews and Meta-analysis extension for Scoping Reviews (PRISMA-ScR) [14, 15].

### Review questions

The Population-Concept-Context framework was used to identify the main concepts in the primary review question and inform the search strategy (Table 1). The primary review question is:

*“Which complex health interventions to prevent early childhood caries are described in the literature*, *how complex are they and what is known about their development*, *evaluation and implementation*?*”* A sub-question to this will be, “*Are there similarities in described and non-described aspects of the development*, *evaluation*, *and implementation of complex health interventions to prevent ECC*?”

**Table 1:** Identifying the main concepts and primary review question using the population-concept-context framework.

**Primary review question:**	Which complex health interventions to prevent early childhood caries are described in the literature, how complex are they and what is known about their development, evaluation, and implementation?
**Population:**	Complex health interventions to prevent early childhood caries that are initiated within the child’s first year of life or during pregnancy.
**Concept:**	1. Identify complex health interventions to prevent early childhood caries. 2. Explore the level of intervention complexity. 3. Explore the identified complex health interventions’ development, evaluation, and implementation. 4. Explore similarities in described and non-described aspects of the development, evaluation and implementation.
**Context:**	All complex health interventions to prevent early childhood caries from all settings and contexts evaluated in a clinical trial are eligible for inclusion.

### Inclusion criteria

#### Population

This review will consider CHI’s to prevent ECC, including pregnant women, parents, and/or primary caregivers. The CHI’s must have started during pregnancy or in the first year of the child’s life and must not be a one-time intervention.

#### Concept

The central concept of this scoping review is to explore the development, evaluation, and implementation of the identified CHI’s to prevent ECC. Therefore this review will extract descriptive data on items regarding the development (i.a., the problem definition, the underlying theory of the intervention, need identification of interventionists or receivers and pilot/feasibility tests), evaluation (i.a., the description of primary and secondary outcomes, cost-effectiveness analysis, process evaluation and intervention fidelity), and implementation (i.a., the reporting on modification and adaptations, training of interventionists and the materials used). For all items, the description level will be examined (i.e. fully described, partially described or non-described), and similarities in described and non-described aspects will be sought in relation to particular study or intervention characteristics, such as the level of intervention complexity. To identify and select suitable CHI’s to be included in this review, the consolidated definition of complex interventions will be used as proposed by Guise et al. (2017) [9].

#### Context

This review will consider interventions from all settings and contexts in which a CHI to prevent ECC is implemented and evaluated. Studies will be included regardless of country of origin or sociocultural setting. The comparison conditions for CHI’s can be usual oral healthcare, no oral healthcare, standard oral health advice (e.g. educational flyers, providing toothbrushes, watching short films), or other interventions with pregnant women or primary caregivers of infants in the first year of life.

#### Types of sources

The first step is identifying CHI’s to prevent ECC initiated during pregnancy or in the first year of life. Only CHI’s evaluated in clinical trials will be included in the screening and selection process. Clinical trials are defined as clinical studies in which participants are assigned to receive one or more interventions so that researchers can evaluate the interventions in terms of biomedical or health-related outcomes [16]. In order to avoid missing any relevant information, all “sibling” publications (i.e. multiple publications on the study or intervention) of an included CHI that describe the development, evaluation, or implementation will be incorporated. These publications can be of all types of study methodology (e.g. qualitative, quantitative, or mixed methodology). However, only the studies published in English will be included.

### Search strategy

The search strategy for this scoping review follows the three-step search strategy recommended by JBI [14]. First, an initial limited search was undertaken in 2019 in PubMed to identify relevant articles. Then, text words contained in the titles and abstracts of relevant articles, the MeSH terms describing the articles, and a search hedge for children (0–4 years) drafted by Ket [17] were used to develop a complete search strategy in consultation with a medical information specialist (KAZ) (S1 File). The databases to be searched include PubMed, Embase, Clarivate Analytics/Web of Science Core Collection, ClinicalTrials.gov, and Wiley/Cochrane Library. The search strategy will be adapted for these databases and other information sources, including all the identified keywords and index terms. No methodological search filter on study design or date that would limit the results will be used in the search strategy. Finally, the reference list of all included studies will be screened for additional CHI’s or sibling publications with additional information about the CHI’s development, evaluation, or implementation. The sources of grey literature to be searched include the registration of included CHI’s in clinical trial registers (e.g. ClinicalTrials.gov).

### Study selection

Following the search, all identified citations will be collated and uploaded into EndNote v.X20.01 (Clarivate Analytics, PA, USA). Duplicates will be removed using the Amsterdam Efficient Deduplication and Bramer methods [18, 19]. Two independent reviewers will perform screening and selection procedures (MLCMO and PCJMvS). ASReview will be used to rank potentially relevant titles and abstracts using an active learning algorithm to determine the threshold for manual screening [20]. Simultaneously, this is the initial pilot test and norm-finding of the title and abstract screening criteria. Subsequently, potentially relevant titles and abstracts will be screened independently by MLCMO and PCJMvS for eligibility using Rayyan [21]. Full-text articles will be screened when judging eligibility criteria based on the title and abstract is impossible. Following a pilot test and norm-finding for the full-text selection criteria of at least two publications, the full text of the selected citations will be assessed in detail against the inclusion criteria by two independent reviewers. The scoping review will record and report the reasons for excluding sources of evidence at the full-text stage that do not meet the inclusion criteria. During the selection process, any disagreements between the reviewers will be resolved through discussion or by an additional reviewer (LCS). The search results and study inclusion process will be reported in the final scoping review and presented in a PRISMA-ScR flow diagram [15].

### Data extraction

Two reviewers (MMCMO and PCJMvS) will perform data charting of the papers included in the scoping review. The reviewers adapted, and pilot tested the JBI data charting template to record essential information about the source (S2 File). The charted data will include specific details about the study, participants, concepts (e.g. type of intervention and control), context (e.g. study setting), methods, and key findings relevant to the review questions. Charting these results may lead to unforeseen but valuable data. Therefore, charting the results will be an iterative process whereby the charting table can be continually updated. Descriptive data will be extracted based on the Intervention Complexity Assessment Tool for Systematic Reviews [22] to describe the complexity of the included CHI’s. In addition, this study will use a 39-item scoring instrument composed of several frameworks for developing and evaluating CHI’s. This scoring instrument covers the phases of the Medical Research Council framework. It will be used to explore the development (including feasibility and piloting), evaluation, and implementation of the included interventions in more depth. This 39-item instrument is based on the scoring instrument developed by Smit et al. (2018) [23]. In recent years, the implementation phase has gained more attention within intervention research, and items covering this phase were lacking in the instrument of Smit et al. (2018) [23]. Therefore, items regarding implementation theories, models, and frameworks used to develop and evaluate CHI’s are added to the instrument [24–28]. A draft extraction form is provided for the adapted instrument (S3 File). The reviewers (MMCMO and PCJMvS) will pilot the draft extraction form with two interventions and modify and revise it as necessary while extracting descriptive data on each of the 39 items from the included evidence sources. It is then assessed whether all items are fully, partially or non-described. The modifications will be detailed in the scoping review. Any disagreements between the reviewers (MMCMO and PCJMvS) will be resolved through discussion or by an additional reviewer (LCS).

### Data analysis and presentation

The presentation of the results will include at least an overview of the identified CHI’s alongside study characteristics and the level of complexity. Furthermore, according to common frameworks, the extent to which CHI’s development, evaluation, and implementation are described according to common frameworks will be visually presented per phase in a co-occurrence matrix. This matrix helps identify gaps and similarities in described and non-described aspects in the description of CHI’s development, evaluation, and implementation. In addition, other visual outputs will be considered during the analysis. Finally, all tables and visual outputs will complement the narrative summary in response to the review questions.

## Discussion

The proposed scoping review aims to map CHI’s to prevent ECC that start during pregnancy or in the first year of life, explore its complexity and the description of relevant aspects of the development, evaluation, and implementation. The outcomes of this scoping review should provide an overview of published complex oral health interventions to prevent ECC, including their level of complexity. Furthermore, gaps may be identified by exploring the development, evaluation and implementation details. Finally, similarities in described and non-described aspects can be made visually in a co-occurrence matrix.

Getting more insight into apparently simple interventions’ level of complexity may create more awareness of the difficulties it brings with them. In addition, the outcomes of this scoping review may provide a direction for the future design of studies on complex interventions to prevent ECC and complete information for those who want to adopt successful interventions.

## Supporting information

S1 Checklist PRISMA-P checklist. (DOCX)

S1 File Initial search strategy developed for Pubmed. (DOCX)

S2 File Data charting template. (DOCX)

S3 File Data extraction form for the 39-item scoring instrument. (DOCX)
