# Supplementary material for: Exploring the development, evaluation and implementation of complex health interventions to prevent early childhood caries in preschool children: A scoping review protocol
Source: PLoS One. 2022 Oct 10;17(10):e0275501. doi: 10.1371/journal.pone.0275501 (PMC9550072; doi:10.1371/journal.pone.0275501)
Supplement: S1 File — (DOCX) [file pone.0275501.s002.docx]

### S1 File: Initial search strategy

| **Search** | **PubMed Query – February 11, 2021** | **Results** |
| --- | --- | --- |
| 4 | #1 AND #2 AND #3 | 6,580 |
| 3 | "Health Education, Dental"[Mesh] OR "Directive Counseling"[Mesh] OR "Index"[tiab] OR "promotion"[tiab] OR "intervention"[tiab] OR "education"[tiab] OR "control"[tiab] OR "guidance"[tiab] OR "prevent*"[tiab] OR "risk"[tiab] OR "directive counseling"[tiab] OR "motivational interviewing"[tiab] OR "anticipatory guidance"[tiab] OR "NOCTP"[tiab] OR "nexo"[tiab] | 6,817,273 |
| 2 | "Dental Caries/prevention and control"[Mesh] OR "Oral Health/education"[Mesh] OR Dental plaque/prevention and control [Mesh] OR "ECC"[tiab] OR "Pufa index"[tiab] OR (("caries"[tiab] OR "cariogenesis"[tiab] OR "carious"[tiab] OR "plaque"[tiab] OR "debris"[tiab] OR "decay"[tiab] OR "Dmft"[tiab] OR "Dmfs"[tiab]) AND ("fissure"[tiab] OR "dental"[tiab] OR "dentin*"[tiab] OR "tooth"[tiab] OR "teeth"[tiab] OR "enamel"[tiab] OR "molar*"[tiab])) | 69,013 |
| 1 | "Infant"[Mesh:NoExp] OR "Infant, Newborn"[Mesh:NoExp] OR "Child, Preschool"[Mesh] OR "Schools, Nursery"[Mesh] OR "infan*"[tiab] OR "pediatri*"[tiab] OR "paediatr*"[tiab] OR "neonat*"[tiab] OR "baby"[tiab] OR "babies"[tiab] OR "toddler*"[tiab] OR "newborn*"[tiab] OR "postneonat*"[tiab] OR "postnat*"[tiab] OR "preschool*"[tiab] OR "pre-school"[tiab] OR "suckling*"[tiab] OR kindergart*[tiab] OR "youngster*"[tiab] OR "young child*"[tiab] OR "kid"[tiab] OR "kids"[tiab] OR "Schools, Nursery"[Mesh] OR "nursery school*"[tiab] OR "playgroup*"[tiab] OR "play-group*"[tiab] OR "playschool*"[tiab] OR "play school"[tiab] OR "child daycare"[tiab] OR "child day care"[tiab] OR ("daycare"[tiab] AND "child*"[tiab]) OR ("day care"[tiab] AND "child*"[tiab]) OR "child care"[tiab] OR "childcare"[tiab] OR "early childhood"[tiab] | 2,222,340 |
