# Supplementary material for: Exploring the development, evaluation and implementation of complex health interventions to prevent early childhood caries in preschool children: A scoping review protocol
Source: PLoS One. 2022 Oct 10;17(10):e0275501. doi: 10.1371/journal.pone.0275501 (PMC9550072; doi:10.1371/journal.pone.0275501)
Supplement: S2 File — (DOCX) [file pone.0275501.s003.docx]

### S2 File: Data charting template

| **Scoping Review Details** | |
| --- | --- |
|  | |
| Scoping Review Title: |  |
| Review’s objectives: |  |
| Review questions |  |
| **Inclusion/Exclusion criteria** | |
| Population: |  |
| Concept: |  |
| Context: |  |
| Types of evidence sources: |  |
| **Evidence source details and characteristics** | |
| Citation details (e.g. author(s), date, title, journal, volume, issue, pages): |  |
| Country: |  |
| Context: |  |
| Participants: |  |
| Start date: |  |
| End date: |  |
| Study funding sources: |  |
| Possible conflicts of interests: |  |
| **Methods** | |
| Aim of the intervention: |  |
| Study design: |  |
| Type of intervention: |  |
| Type of control intervention: |  |
| Intervention description: |  |
| **Participants** | |
| Population description: |  |
| Inclusion criteria: |  |
| Exclusion criteria: |  |
| Method of recruitment of participants: |  |
| Total number of participants: |  |
| **Results** | |
| Outcome measures: |  |
| Outcomes and effects: |  |
