# Supplementary material for: Exploring the development, evaluation and implementation of complex health interventions to prevent early childhood caries in preschool children: A scoping review protocol
Source: PLoS One. 2022 Oct 10;17(10):e0275501. doi: 10.1371/journal.pone.0275501 (PMC9550072; doi:10.1371/journal.pone.0275501)
Supplement: S3 File — (DOCX) [file pone.0275501.s004.docx]

### S3 File: Data extraction form 39-item scoring instrument – Scoping instrument on development, evaluation and implementation, based on Smit et al. 2018

| **MRC stage** | **Item number** | **Item** | **Brief description** | **References** |
| --- | --- | --- | --- | --- |
| **Development** | 1 | Was the problem clearly defined? | Did the problem definition match the proposed intervention? | ^1,2^ |
|  | 2 | Was the intervention goal clearly defined? |  | ^3-5^ |
|  | 3 | Was there support in the literature? | Was there use of systematic reviews or other high levels of evidence that (1) underpinned the choice of the intervention and (2) allowed the choice of intervention components in greater depth? | ^1,3-5^ |
|  | 4 | Was there an underlying theory of the proposed intervention? | Which theory underpinned the intervention? What was the rationale for this theory? | ^1,5-7^ |
|  | 5 | Was the content of the intervention described? | What was the content, and when and how was the intervention delivered? | ^3-7^ |
|  | 6 | Duration (total) and intensity of intervention | how many hours were indicated for home visits, training, and meetings, and what was the number of follow-up visits? | ^7^ |
|  | 7 | Identification of the needs of the interventionist and receiver | Were the (care) needs, perceptions, and capacities of intervention recipients and/or interventionists regarding the identified problem and proposed solution/intervention elucidated during the development phase? | ^1^ |
|  | 8 | Who delivered the intervention? | Were the interventionists and their backgrounds and characteristics described? | ^1,3,4,7^ |
|  | 9 | Who received the intervention? | Were the study population and the inclusion and exclusion criteria clearly described? | ^1,3,4^ |
|  | 10 | Were contextual factors examined in advance? | Has a current practice analysis been conducted? Were factors examined in advance to prevent a misfit between the intervention and current practice? | ^1,7,8^ |
|  | 11 | Were potential barriers and facilitators regarding the delivery or content of the intervention identified? | Were potential barriers and facilitators of the intervention identified in advance? | ^5,6^ |
|  | 12 | Was there modelling of process and outcome procedures/phases? | How was the information from the literature synthesized and mapped? Was the choice of active components clear? Was any modelling scenario used for this purpose/roadmap? | ^3,4,9,10^ |
|  |  |  |  |  |
|  | 13 | Was a feasibility/pilot study conducted? | The pilot test to determine the feasibility, acceptability, and practicability should have taken into account the key uncertainties that had been identified during the development process. | ^1,3,4,11,12^ |
|  | 14 | Were the results of the pilot study described? | Information on how the intervention was tested. For detailed information. | ^5,6,11^ |
|  |  |  |  |  |
| **Evaluation** | 15 | Was the study design described? | Which design, such as RCT, IRT, or CRT, was used and why? | ^3,4^ |
|  | 16 | Were the primary outcomes described? | What outcomes are most important, and how did the authors deal with multiple outcomes in the analysis? | ^3,4^ |
|  | 17 | Were secondary outcomes described? | What were secondary, and how did the authors deal with multiple outcomes in the analysis? | ^3,4^ |
|  | 18 | Were outcomes in subgroups described? | Did the authors consider which sources of variation in outcomes are important, and did they carry out appropriate subgroup analysis? | ^3,4^ |
|  | 19 | Was a cost-effectiveness analysis conducted? | An economic evaluation should be included if possible, as this will make the results far more useful for decision-makers. | ^4,6^ |
|  | 20 | Are the results of the cost-effectiveness analysis described? | Did the authors describe all costs or required resources for delivering the intervention? | ^3-6^ |
|  | 21 | Was fidelity described? | Quantify the extent to which the intervention was delivered as planned to the target group in terms of the following aspects: coverage of the intervention, how often the intervention was delivered, and the intervention's duration. | ^5,7,13,14^ |
|  | 22 | Was recruitment described? | What planned and actual recruitment procedures were used? | ^13,15^ |
|  | 23 | Was the context described? | Where was the intervention performed? Describe the barriers and facilitators identified during the process evaluation that may have influenced the intervention outcomes. | ^7,13,15^ |
|  | 24 | Was retention of participants described? | How were the participants continuously maintained concerning data collection? | ^15^ |
|  | 25 | The dose received (target group) | To what extent were all of the intended methods, strategies and/or activities used? To what extent were participants present at interventions activities engaged in the activities? How did participants react to specific aspects of the intervention? | ^13^ |
|  | 26 | The dose received (satisfaction target group) | How did participants experience the intervention? Did the intervention satisfy the participants? How was this measured? | ^13^ |
|  | 27 | Dose received (satisfaction research group) | How did the researchers experience the intervention? Did it meet their expectations? | ^13^ |
|  | 28 | Intervention reached | Quantify the proportion of the target population that participated in the intervention. Describe the subjects who did and did not participate. Were the included participants represented? | ^13,15^ |
|  | 29 | Was there a description of all the (training) materials and tools during implementation? | Were the materials and tools described? Was there (open) access available? | ^5-7,13^ |
| **Implementation** | 30 | Adaptations and modifications reported | Why, when, how, what, and what delivery level was modified in the implementation process?  Was the modification planned/proactive or unplanned/reactive? Who determined that the modification should be made? | ^16^ |
|  | 31 | Understanding and explaining what influences implementation outcomes | **Determinant frameworks**  Describe general types of hypothesized determinants that have been found to influence implementation outcomes.  **Implementation theories** Classic theories Apply theories from other fields such as psychology, sociology and organizational theory. | ^17^ |
|  | 32 | Evaluating implementation outcomes | Evaluation frameworks A category of frameworks provides a structure for evaluating implementation endeavors. | ^17^ |
|  | 33 | Was the method of the training described |  | ^7^ |
|  | 34 | Was the content of the training described? |  | ^7^ |
|  | 35 | Was the individual who provided the training described? |  | ^7^ |
|  | 36 | Was the duration and intensity of the training described? |  | ^7^ |
|  | 37 | Did the interventionists undergo an assessment or exam after the training? |  | ^7^ |
|  | 38 | Were the test results described? |  | ^7^ |
|  | 39 | Was the training evaluated? |  | ^7^ |

**References supplement S3**

1. van Meijel B, Gamel C, van Swieten-Duijfjes B, Grypdonck MHF. The development of evidence-based nursing interventions: Methodological considerations. J Adv Nurs. 2004;48(1):84-92.
2. Campbell NC, Murray E, Darbyshire J, et al. Designing and evaluating complex interventions to improve health care. BMJ. 2007;334(7591):455-459.
3. Craig P, Dieppe P, Macintyre S, Michie S, Nazareth I, Petticrew M. Developing and evaluating complex interventions: The new medical research council guidance. BMJ. 2008;337(7676):29-983.
4. Skivington K, Matthews L, Simpson SA, Craig P, Baird J, Blazeby JM, *et al.* A new framework for developing and evaluating complex interventions: Update of medical research council guidance. BMJ. 2021;374:n2061.
5. Möhler R, Bartoszek G, Köpke S, Meyer G. Proposed criteria for reporting the development and evaluation of complex interventions in healthcare (CReDECI): guideline development. Int J Nurs Stud. 2012;49(1):40-6.
6. Möhler R, Köpke S, Meyer G. Criteria for reporting the development and evaluation of complex interventions in healthcare: Revised guideline (CReDECI 2). Trials. 2015;16(1):204.
7. Hoffmann TC, Glasziou PP, Boutron I, Milne R, Perera R, Moher D, *et al.* Better reporting of interventions: Template for intervention description and replication (TIDieR) checklist and guide. BMJ. 2014;348:g1687.
8. Moore GF, Audrey S, Barker M, Bond L, Bonell, C, Hardeman W, *et al.* Process evaluation of complex interventions: Medical research council guidance. BMJ. 2015;350:h1258.
9. Sermeus, W. Modelling process and outcomes in complex interventions. In: Richards DA, Rahm Hallberg I. Complex interventions in health: An overview of research methods. Routledge, London 2015: 111.
10. Campbell M, Fitzpatrick R, Haines A, Kinmonth AL, Sandercock D, Spiegelhalter P, *et al.* Framework for design and evaluation of complex interventions to improve health. BMJ. 2000;321(7262):694-696.
11. Thabane L, Ma J, Chu R, Cheng J, Ismaila A, Rios LP, *et al*. A tutorial on pilot studies: The what, why and how. BMC Med Res Methodol. 2010;10(1):1.
12. Craig P. A new CONSORT extension should improve the reporting of randomized pilot and feasibility trials. J Clin Epidemiol. 2017;84:30-32.
13. Saunders RP, Evans MH, Joshi P. Developing a process-evaluation plan for assessing health promotion program implementation: A how-to guide. Health Promot Practic. 2005;6(2):134-147.
14. Carroll C, Patterson M, Wood S, Booth A, Rick J, Balain S. A conceptual framework for implementation fidelity. *I*mplementation Sci. 2007;2(1):40.
15. Baranowski T, Stables G. Process evaluations of the 5-a-day projects. Health Educ Behav. 2000;27(2):157-166.
16. Wiltsey Stirman S, Baumann AA, Miller CJ. The FRAME: An expanded framework for reporting adaptations and modifications to evidence-based interventions. Implementation Sci. 2019;14(1):58.
17. Nilsen P. Making sense of implementation theories, models and frameworks. Implementation Sci. 2015;10(1):53.
